# Supplementary material for: Unprecedented yet gradual nature of first millennium CE intercontinental crop plant dispersal revealed in ancient Negev desert refuse
Source: eLife. 2023 Nov 27;12:e85118. doi: 10.7554/eLife.85118 (PMC10846859; doi:10.7554/eLife.85118)
Supplement: Supplementary file 1. [file elife-85118-supp1.docx]

Supplementary Table 1. Carpological^[[1]](#footnote-1)^ plant remains from Negev Highland middens

| **Category** | **Latin name** | **Common name** |
| --- | --- | --- |
| **Cereals** | *Hordeum vulgare* subsp. *hexastichum* (hulled) | six-row hulled barley |
|  | *Hordeum vulgare* subsp*. distichum* (hulled) | two-row hulled barley |
|  | *Triticum turgidum* s.l. (free-threshing) | free-threshing tetraploid wheat |
|  | *Triticum aestivum* (free-threshing) | free-threshing hexaploid wheat |
| **Legumes** | *Lens culinaris* | lentil |
|  | *Vicia ervilia* | bitter vetch |
|  | *Vicia faba* | broad beans |
|  | *Lathyrus clymenum* | Spanish vetchling |
|  | *Lupinus* *albus* | white lupine |
|  | *Trigonella foenum-graecum* | fenugreek |
| **Fruits** | *Vitis vinifera* | common grape |
|  | *Ficus carica* | common fig |
|  | *Phoenix dactylifera* | date palm |
|  | *Olea europaea* | European olive |
|  | *Punica granatum* | pomegranate |
|  | *Ceratonia siliqua* | carob |
|  | *Prunus persica* | peach |
|  | *Prunus* subgen*. Cerasus/Prunus* | plum/cherry |
|  | *Ziziphus jujuba/mauritiana* | jujube |
| **Nuts** | *Prunus amygdalus* | almond |
|  | *Pinus pinea* | stone pine |
|  | *Pistacia vera* | pistachio |
|  | *Juglans regia* | Persian walnut |
| **Vegetable** | *Solanum melongena* | aubergine |
| **Wild** | *Vachellia nilotica^[[2]](#footnote-2)^* | Nile acacia |
|  | *Adonis dentata* | toothed pheasant's eye |
|  | *Aizoon hispanicum* | Spanish aizoon |
|  | *Ajuga iva* | herb ivy |
|  | *Ammi majus/visnaga* | bishop's weed |
|  | *Anagallis arvensis* | scarlet pimpernel |
|  | *Anagyris foetida* | Mediterranean stinkbush |
|  | *Andrachne telephioides* | bastard orpine |
|  | *Anthemis pseudocotula* | chamomile |
|  | *Arnebia decumbens* | Arabian primrose |
|  | *Asphodelus tenuifolius/fistulosus* | onionweed |
|  | *Astragalus hamosus/arpilobus* | milkvetch |
|  | *Atriplex glauca* | waxy saltbush |
|  | *Avena barbata* | slender wild oat |
|  | *Avena sterilis* | animated oat |
|  | *Bassia muricata* | smotherweed |
|  | *Bellevalia* sp. | Roman squill |
|  | *Beta vulgaris* | beet |
|  | *Bifora testiculata* | European bishop |
|  | *Brachypodium distachyon* | purple false brome |
|  | *Bromus* type | brome (type) |
|  | *Buglossoides tenuiflora* | corn gromwell |
|  | *Bupleurum lancifolium* | lanceleaf thorow wax |
|  | *Calendula* sp. | calendula |
|  | *Cardaria draba* | hoary cress |
|  | *Carrichtera annua* | Ward’s weed |
|  | *Carthamus* sp. | thistle |
|  | *Caylusea hexagyna* |  |
|  | *Centaurea* sp. | knapweed |
|  | *Cephalaria joppensis* | Jaffa scabious |
|  | *Chenopodium murale* | nettleleaf goosefoot |
|  | *Cichorium endivia* | endive |
|  | *Citrullus colocynthis* | colocynth |
|  | *Convolvulus cf. arvensis* | bindweed |
|  | *Coriandrum sativum* | coriander |
|  | *Coronilla* cf*. repanda* |  |
|  | cf. Crassula/Sedum | stonecrops |
|  | *Cutandia memphitica/dichotoma* | cutandia grass |
|  | *Cynodon dactylon* | Bermuda grass |
|  | *Daucus/Torilis* | wildcarrot/hedgeparsley |
|  | *Echiochilon fruticosum* | bushy bugloss |
|  | *Echium* cf. *angustifolium* | bugloss |
|  | *Emex spinosa* | devil's thorn |
|  | *Erucaria microcarpa* | pink mustard |
|  | *Erucaria pinnata* | pink mustard |
|  | *Euphorbia falcata* | sickle spurge |
|  | *Fagonia* sp. | fagonbush |
|  | *Fumaria parviflora* | fineleaf fumitory |
|  | *Galium aparine* | cleavers |
|  | *Gastrocotyle hispida* | hairy bugloss |
|  | *Glaucium arabicum* | horned poppy |
|  | *Glebionis coronaria* | garland chrysanthemum |
|  | *Gypsophila capillaris* | desert baby's breath |
|  | *Gypsophila pilosa* | Turkish baby's breath |
|  | *Haplophyllum* cf*. tuberculatum* | plant of the mosquito |
|  | *Hedysarum spinosissimum* | spiny sulla |
|  | *Heliotropium* sp. | heliotrope |
|  | *Hippocrepis unisiliquosa* | common horseshoe vetch |
|  | *Hordeum glaucum* | wall barley |
|  | *Hordeum marinum/hystrix* | sea/Mediterranean barley |
|  | *Hordeum vulgare* subsp*. spontaneum* | wild barley |
|  | *Hyoscyamus reticulatus* | henbane |
|  | cf. *Lathyrus aphaca* | yellow vetchling |
|  | *cf. Lathyrus blepharicarpos* | ciliate vetchling |
|  | *Lathyrus hierosolymitanus* | Jerusalem vetchling |
|  | *Lathyrus marmoratus* cf*. Vicia narbonensis* | vetchling cf. purple broad bean |
|  | *Lathyrus* sect. *cicercula* | vetchling |
|  | cf. *Lavandula coronopifolia* | stagshorn lavender |
|  | Lithospermeae |  |
|  | *Lolium rigidum* | rigid ryegrass |
|  | *Lolium temulentum* | darnel ryegrass |
|  | cf. *Lotus peregrinus* | bird's foot trefoil |
|  | *Malva aegyptia* | Egyptian mallow |
|  | *Malva parviflora* | cheeseweed mallow |
|  | *Medicago astroites* | medick |
|  | *Medicago polymorpha/marina* | bur clover/sea medick |
|  | *Medicago tuberculata* | medick |
|  | *Melilotus sulcatus* | furrowed melilot |
|  | *Mesembryanthemum nodiflorum* | slenderleaf iceplant |
|  | *Moltkiopsis ciliata* | callous-leaved gromwell |
|  | *Neslia apiculata* | ball mustard |
|  | *Nonea echioides/melanocarpa* | monkswort |
|  | *Papaver* sp. | poppy |
|  | *Peganum harmala* | wild rue |
|  | *Phalaris minor* | small canary grass |
|  | *Phalaris paradoxa* | Mediterranean canary grass |
|  | *Picris* sp. | oxtongue |
|  | *cf. Pinus halepensis* | cf. Aleppo pine |
|  | *Pistacia atlantica* | atlas pistachio |
|  | *Plantago chamaepsyllium/notata* | plantain |
|  | *Plantago* *ovata* | blond plantain |
|  | *Pteranthus dichotomus* |  |
|  | *Pulicaria incisa* |  |
|  | *Raphanus raphanistrum* | wild radish |
|  | *Rapistrum rugosum* | annual bastardcabbage |
|  | *Reseda muricata* | mignonette |
|  | *cf. Rhus coriaria* | cf. elm-leaved sumach |
|  | *Rumex* sp*.* | dock |
|  | Salsoleae | saltwort |
|  | *Scorpiurus muricatus* | prickly scorpion's-tail |
|  | *Silene colorata/decipiens* | catchfly |
|  | *Solanum villosum/nigrum* | hairy/black nightshade |
|  | *Spergula fallax* | spurry |
|  | *Suaeda* sp. | seepweed |
|  | *Tamarix aphylla* | athel tamarisk |
|  | *Teucrium capitatum* | cat-thyme germander |
|  | *Thesium humile/bergeri* | bastard toadflax |
|  | *Thymelaea* cf. *passerina/gussonei* | mezereon/sparrow-wort |
|  | *Thymelaea hirsuta* | shaggy sparrow-wort |
|  | *Trifolium campestre/glanduliferum* | field/glandular clover |
|  | *Trifolium* sp. | clover |
|  | *Trigonella arabica* | Arabian fenugreek |
|  | *Vaccaria hispanica* | cow cockle |
|  | *Verbascum* sp. | mullein |
|  | *Vicia hybrida/sericocarpa* | vetch |
|  | *Vicia palaestina/sativa* | Palestine/common vetch |
|  | *Vicia peregrina/narbonensis* | wandering/purple broad vetch |
|  | *Vicia sativa* | common vetch |
|  | *Vicia villosa/tenuifolia* | hairy/fine-leaved vetch |
|  | *Zilla spinosa* | spiny zilla |

1. Includes taxa identified by other preserved plant parts, e.g. perianth, rachis fragments, segmented stems/leaves. Nomenclature follows http://flora.org.il. [↑](#footnote-ref-1)
2. We take this Egyptian wild plant to have been cultivated or imported into the Negev Highlands (see Results). [↑](#footnote-ref-2)
