# Supplementary material for: Unprecedented yet gradual nature of first millennium CE intercontinental crop plant dispersal revealed in ancient Negev desert refuse
Source: eLife. 2023 Nov 27;12:e85118. doi: 10.7554/eLife.85118 (PMC10846859; doi:10.7554/eLife.85118)
Supplement: Supplementary file 2. [file elife-85118-supp2.docx]

Supplementary Table 2. Presence/absence of domesticated species in Negev Highland middens by period (carpological remains)

| **Plants/centuries CE** | |  | **1^st^–3^rd^** | **4^th^–mid-5^th^** | **mid-5^th^–mid-6^th^** | **mid-6^th^–mid-7^th^** | **7^th^** | **mid-7^th^–8^th^** |
| --- | --- | --- | --- | --- | --- | --- | --- | --- |
| **Functional category** | **Latin name** | **Common name** |  |  |  |  |  |  |
| **Cereals** | *Hordeum vulgare* | Barley | ✓ | ✓ | ✓ | ✓ | ✓ | ✓ |
|  | *Triticum turgidum* s.l. | Free-threshing tetraploid wheat | ✓ | ✓ | ✓ | ✓ | ✓ | ✓ |
|  | *Triticum aestivum* | Free-threshing hexaploid wheat |  |  |  | ✓ | ✓ | ✓ |
| **Legumes** | *Lens culinaris* | Lentil | ✓ | ✓ | ✓ | ✓ | ✓ | ✓ |
|  | *Vicia ervilia* | Bitter vetch | ✓ | ✓ | ✓ | ✓ | ✓ | ✓ |
|  | *Vicia faba* | Broad bean |  |  |  |  | ✓ | ✓ |
|  | *Lathyrus clymenum* | Spanish vetchling |  |  |  |  | ✓ | ✓ |
|  | *Lupinus albus* | White lupine |  |  |  |  | ✓ |  |
|  | *Trigonella foenum-graecum* | Fenugreek |  | ✓ |  |  | ✓ | ✓ |
| **Fruits** | *Vitis vinifera* | Grape | ✓ | ✓ | ✓ | ✓ | ✓ | ✓ |
|  | *Ficus carica* | Fig | ✓ | ✓ | ✓ | ✓ | ✓ | ✓ |
|  | *Olea europaea* | Olive | ✓ | ✓ | ✓ | ✓ | ✓ | ✓ |
|  | *Phoenix dactylifera* | Date | ✓ | ✓ | ✓ | ✓ | ✓ | ✓ |
|  | *Punica granatum* | Pomegranate |  | ✓ | ✓ | ✓ | ✓ | ✓ |
|  | *Ceratonia siliqua* | Carob |  | ✓ |  | ✓ | ✓ | ✓ |
|  | *Prunus persica* | Peach |  | ✓ |  | ✓ | ✓ | ✓ |
|  | *Prunus* subgen*. Cerasus/Prunus* | Plum/cherry |  |  |  |  |  | ✓ |
|  | *Ziziphus jujuba/mauritiana* | Jujube |  |  |  |  |  | ✓ |
| **Nuts** | *Prunus amygdalus* | Almond |  |  |  | ✓ | ✓ | ✓ |
|  | *Pinus pinea* | Stone pine |  |  |  |  | ✓ | ✓ |
|  | *Pistacia vera* | Pistachio |  |  |  |  |  | ✓ |
|  | *Juglans regia* | Walnut |  |  |  |  |  | ✓ |
| **Vegetable** | *Solanum melongena* | Aubergine |  |  |  |  | ✓ | ✓ |
| **Other** | *Vacchelia nilotica* | Nile acacia |  | ✓ | ✓ |  |  |  |
