# Supplementary material for: Unprecedented yet gradual nature of first millennium CE intercontinental crop plant dispersal revealed in ancient Negev desert refuse
Source: eLife. 2023 Nov 27;12:e85118. doi: 10.7554/eLife.85118 (PMC10846859; doi:10.7554/eLife.85118)
Supplement: Supplementary file 3. [file elife-85118-supp3.docx]

Supplementary Table 3. Identified wood and charcoal taxa from Shivta, Nessana^[[1]](#footnote-1)^ and Elusa^[[2]](#footnote-2)^

| Category | Taxon | English common name | SVT | NZN | HLZ |
| --- | --- | --- | --- | --- | --- |
| Fruit trees | *Ficus carica* | common fig | + | + | + |
|  | *Ficus sycomorus* | Sycomore fig | - | + | + |
|  | *Hyphaene thebaica* | doum palm | + | + | - |
|  | *Olea europaea* | olive | + | - | + |
|  | *Phoenix dactylifera* | date palm | + | - | - |
|  | *Prunus* spp. (*dulcis/armeniaca*) | plum/apricot | + | + | - |
|  | *Punica granatum* | pomegranate | - | + | - |
|  | *Vitis vinifera* | grapevine | + | + | - |
| Exotic trees | *Buxus sempervirens* | boxwood | + | + | - |
|  | *Cedrus libani* | cedar of Lebanon | + | + | - |
|  | *Fraxinus excelsior* | European ash | - | + | - |
| Desert trees and shrubs | *Calotropis procera* | apple of Sodom | + | + | - |
|  | *Capparis spinosa* | caper bush | + | - | - |
|  | *Fagonia mollis* | fagonia | - | + | - |
|  | *Juniperus phoenicea* | Phoenician juniper | + | + | - |
|  | *Lycium* spp. | boxthorn | + | + | + |
|  | *Moringa peregrina* | Ben tree | + | - | - |
|  | *Pistacia atlantica* | Persian turpentine | + | + | - |
|  | *Populus/Salix* | poplar/willow | - | + | - |
|  | *Retama raetam* | white broom | + | + | + |
|  | *Rhamnus* spp. | buckthorn | + | + | + |
|  | *Salsola tetrandra* | saltwort [tetrandra] | + | - | - |
|  | *Salsola vermiculata* | Mediterranean saltwort | + | + | - |
|  | *Tamarix* spp. | tamarisk | + | + | + |
|  | *Ziziphus/Paliurus* | jujube/Jerusalem thorn | + | + | + |
|  | *Zygophyllum dumosum* | bushy bean caper | + | + | - |
| Mediterranean trees and shrubs | *Crataegus* spp. | hawthorn group/Maloideae | + | + | + |
|  | *Cupressus sempervirens* | Italian cypress | + | + | + |
|  | *Myrtus communis* | true myrtle | - | + | - |
|  | *Pinus halepensis* | Aleppo pine | + | + | + |
|  | *Pistacia palaestina* | terebinth | + | + | + |
|  | *Platanus orientalis* | oriental plane | - | + | + |
|  | *Quercus calliprinos* | Kermes oak | + | - | + |
|  | *Vitex agnus-castus* | chaste tree | - | + | - |

1. Data for Shivta and Nessana derive from Table 1 in: Langgut, D., et al. (2021) Environment and horticulture in the Byzantine Negev Desert, Israel: Sustainability, prosperity and enigmatic decline. *Quaternary International*, 593: 160–177 [↑](#footnote-ref-1)
2. Data for Elusa are based on Table S8 in: Bar-Oz, G., et al. (2019) Ancient trash mounds unravel urban collapse a century before the end of Byzantine hegemony in the southern Levant. *PNAS*, 116(17): 8239–8248. [↑](#footnote-ref-2)
