# Supplementary material for: Unprecedented yet gradual nature of first millennium CE intercontinental crop plant dispersal revealed in ancient Negev desert refuse
Source: eLife. 2023 Nov 27;12:e85118. doi: 10.7554/eLife.85118 (PMC10846859; doi:10.7554/eLife.85118)
Supplement: Supplementary file 4. [file elife-85118-supp4.docx]

Supplementary Table 4. Identified pollen from Shivta reservoirs and garden

| **Taxon** | **English common name** | **S reservoir** | **N reservoir** | **N church** |
| --- | --- | --- | --- | --- |
| *Artemisia* | sagebrush | + | + | + |
| *Asphodelus* | asphodels | - | + | + |
| Asteraceae Asteroideae type | aster-like | + | + | + |
| Asteraceae Cichorioideae type | dandelion-like | + | + | + |
| Brassicaceae | mustards | + | + | + |
| *Bunium* type | umbellifers | + | + | + |
| *Calendula* | marigold | + | - | - |
| *Carduus* | plumeless thistles | - | - | - |
| *Carthamus* | distaff thistle | + | - | + |
| Caryophyllaceae | pinks | - | + | + |
| *Cedrus* | cedar | + | + | + |
| *Centaurea* | knapweeds | - | + | + |
| *Ceratonia siliqua* | carob | + | - | + |
| Cerealia | cereals | + | + | + |
| Chenopodiaceae | chenopods | + | + | + |
| *Cistus* | rock rose | + | + | - |
| *Corylus* | hazel | + | - | + |
| *Crocus* | crocus | - | + | - |
| Cyperaceae | sedges | + | + | + |
| *Ephedra* | Mormon-tea | + | + | + |
| Fabaceae | legumes | + | + | + |
| *Ferula* type |  | - | + | + |
| *Fraxinus* | ash | + | + | + |
| *Geranium* | cranesbill | + | + | + |
| Juniperus/Cupressus | juniper/cypress | + | + | + |
| *Lemna* | duckweeds | - | + | - |
| Liliaceae | lilies | + | + | + |
| Malvaceae | mallows | - | + | + |
| *Myrtus communis* | true myrtle | - | - | + |
| *Nymphaea* | water lilies | + | + | + |
| *Olea europaea* | olive | + | + | + |
| *Phoenix dactylifera* | date palm | + | + | + |
| Pinaceae | pine family | - | + | + |
| *Pinus* | pine | + | + | + |
| Plantaginaceae | plantains | + | + | + |
| Poaceae | grasses | + | + | + |
| Polygonaceae | knotweeds | + | + | + |
| *Potamogeton* | pondweed | - | + | - |
| Ranunculaceae | buttercup | - | + | - |
| *Rumex* | docks | - | - | + |
| *Salix* | willow | + | + | + |
| *Scilla* | squills | - | + | - |
| *Sparganium* | bur-reeds | - | - | + |
| *Tamarix* | tamarisk | - | - | + |
| Thymelaeaceae | sparrow-wort | + | + | + |
| *Ulmus* | elm | - | + | - |
| *Vitis vinifera* | grapevine | + | + | + |
| *Zygophyllum* | bean-caper | - | - | + |
