# Supplementary material for: Unprecedented yet gradual nature of first millennium CE intercontinental crop plant dispersal revealed in ancient Negev desert refuse
Source: eLife. 2023 Nov 27;12:e85118. doi: 10.7554/eLife.85118 (PMC10846859; doi:10.7554/eLife.85118)
Supplement: Supplementary file 5. [file elife-85118-supp5.docx]

Supplementary Table 5. Combined evidence for fruit/nut trees

| **Taxon** | **Common name** | **Seeds/Fruit** | | | **Charcoal** | | | **Pollen** | | | |
| --- | --- | --- | --- | --- | --- | --- | --- | --- | --- | --- | --- |
|  |  | *SVT* | *NZN* | *HLZ* | *SVT* | *NZN* | *HLZ* | *SVT 1* | *SVT 2* | *SVT 3* |  |
| *Vitis vinifera* | grapevine | + | + | + | + | + | - | + | + | + |  |
| *Olea europaea* | olive | + | + | + | + | - | + | + | + | + |  |
| *Ficus carica* | common fig | + | + | + | + | + | + | - | - | - |  |
| *Phoenix dactylifera* | date palm | + | + | + | + | - | - | + | + | + |  |
| *Ceratonia siliqua* | carob | + | + | + | - | - | - | + | - | + |  |
| *Punica granatum* | pomegranate | + | + | + | - | + | - | - | - | - |  |
| *Prunus* spp. | almond/peach/plum | + | + | + | + | + | - | - | - | - |  |
| *Pinus* spp. | pine | + | + | - | + | + | + | + | + | + |  |
| *Corylus* sp. | hazel | - | - | - | - | - | - | + | - | + |  |
| *Ficus sycomorus* | sycomore fig | - | - | - | - | + | + | - | - | - |  |
| *Hyphaene thebaica* | doum palm | - | - | - | + | + | - | - | - | - |  |
| *Juglans regia* | walnut | + | - | - | - | - | - | - | - | - |  |
| *Pistacia vera* | pistachio | + | - | - | * | * | - | - | - | - |  |
| *Ziziphus jujuba/mauritiana* | jujube | + | - | - | * | * | - | - | - | - |  |

Carpological, anthracological and palynological evidence for fruit/nut trees in the study sites. Assessment of local cultivation is based on the combination of proxies and especially pollen to include grapevine, fig, olive, date, pomegranate, carob, hazel, cedar and the *Prunus* genus (potentially including almond, peach, plum and/or cherry). Local cultivation of stone pine may also plausibly be inferred. SVT1= South reservoir, Shivta; SVT 2 = North reservoir, Shivta; SVT3 = North church garden, Shivta; + indicates presence; - indicates absence; *indicates charcoal identified to genus, including possible local wild species. *Prunus* spp. includes *Prunus amygdalus* (syn. *P. dulcis*) and *Prunus domestica/cerasus* endocarp/exocarp, as well as *Prunus* spp. charcoal. *Pinus* spp. includes *Pinus pinea* seed coats, *Pinus halepensis* charcoal, and *Pinus* sp. pollen.
