# Supplementary material for: Unprecedented yet gradual nature of first millennium CE intercontinental crop plant dispersal revealed in ancient Negev desert refuse
Source: eLife. 2023 Nov 27;12:e85118. doi: 10.7554/eLife.85118 (PMC10846859; doi:10.7554/eLife.85118)
Supplement: Supplementary file 6. [file elife-85118-supp6.docx]

#### Supplementary Table 6. Radiocarbon dating of select loci

| **Radiocarbon Lab. no.** | **Site** | **Locus** | **Basket** | **Special find** | **Material dated** | **Uncal BP** | **Cal. CE (1 σ)** | **Cal. CE (2 σ)** |
| --- | --- | --- | --- | --- | --- | --- | --- | --- |
| Poz-141223 | Nessana | 101 | 1040-1 | white lupine | charred barley seed | 1335 ± 30 | 654 (46.4%) 682 | 647 (61.0%) 708 |
|  |  |  |  |  |  |  | 745 (18.3%) 760 | 730 (34.5%) 775 |
|  |  |  |  |  |  |  | 768 ( 3.6%) 771 |  |
| Poz-141225 | Shivta | 504 | 5029 | aubergine | charred barley seed | 1170 ± 30 | 776 ( 9.7%) 788 | 772 (73.9%) 901 |
|  |  |  |  |  |  |  | 825 (49.0%) 894 | 916 (21.6%) 974 |
|  |  |  |  |  |  |  | 928 ( 9.6%) 945 |  |
| Poz-141226 | Shivta | 501 | 5108 | jujube | charred barley seed | 1295 ± 30 | 670 (33.4%) 704 | 659 (95.4%) 775 |
|  |  |  |  |  |  |  | 739 (34.9%) 772 |  |

Radiocarbon dating was performed by the Poznan Radiocarbon Laboratory, and calibration was made with the OxCal v4.4.2 (Bronk Ramsey 2020), using atmospheric data from Reimer et al (2020).

References:

Bronk Ramsey, C. 2020. OxCal v4.4.2. Available at: <https://c14.arch.ox.ac.uk/oxcal/OxCal.html>

Reimer, P. et al. 2020. The IntCal20 Northern Hemisphere radiocarbon age calibration curve (0–55 cal kBP). *Radiocarbon* 62: 725–57.<https://doi.org/10.1017/RDC.2020.41>
