# Supplementary material for: Unprecedented yet gradual nature of first millennium CE intercontinental crop plant dispersal revealed in ancient Negev desert refuse
Source: eLife. 2023 Nov 27;12:e85118. doi: 10.7554/eLife.85118 (PMC10846859; doi:10.7554/eLife.85118)
Supplement: Supplementary file 7. [file elife-85118-supp7.docx]

Supplementary Table 7. Earliest archaeobotanical evidence in the S Levant for domestication/introduction of Negev Highland domesticated plants

| **Tag** | **Latin name** | **English common name** | **Period** | **Approx date** | **Reference** |
| --- | --- | --- | --- | --- | --- |
| Neolithic domesticate | *Hordeum vulgare* | barley | PPNB | 9^th^ mill. BCE | Zohary et al. 2012 |
|  | *Lens culinaris* | lentil | PPNB | 9^th^ mill. BCE | Caracuta et al. 2017 |
|  | *Vicia ervilia* | bitter vetch | PPNB | 9^th^ mill. BCE | Caracuta et al. 2017 |
|  | *Vicia faba* | broad bean | PPNB | 9^th^ mill. BCE | Caracuta et al. 2017 |
|  | *Triticum turgidum* s.l. (free-threshing) | durum wheat (s.l.) | PPNB | 7^th^ mill. BCE | Feldman and Kislev 2007 |
|  | *T. aestivum* (free-threshing) | bread wheat (s.l.) | NA | NA | Zohary et al. 2012 |
| Early fruit domesticate | *Olea europaea* | olive | Chalcolithic | 5^th^ mill. BCE | Langgut et al. 2019 |
|  | *Ficus carica* | fig | Chalcolithic | 5^th^ mill. BCE | Weiss 2015 |
|  | *Vitis vinifera* | grape | Chalcolithic | 5^th^ mill. BCE | Weiss 2015 |
|  | *Phoenix dactylifera* | date | Chalcolithic | 5^th^ mill. BCE | Weiss 2015 |
|  | *Punica granatum* | pomegranate | Chalcolithic | 5^th^ mill. BCE | Melamed 2002 |
|  | *Prunus amygdalus* | almond | Chalcolithic | 5^th^ mill. BCE | Zohary et al. 2012 |
| Bronze-Iron Age introduction | *Lathyrus clymenum* | Spanish vetchling | Middle Bronze | 19^th^-18^th^ c. BCE | Kislev et al. 1993 |
|  | *Juglans regia* | walnut | Middle Bronze | 18^th^ c. BCE | Langgut 2015 |
|  | *Trigonella foenum-graecum* | fenugreek | Late Bronze Age IIA | 14^th^ c. BCE | Weiss et al. 2019 |
| RAD crop | *Prunus persica* | peach | Nabatean | 1^st^ c. BCE | Kislev and Simchoni 2009 |
|  | *Vachellia nilotica* | Nile acacia | Nabatean | 1^st^ c. BCE | Kislev 1990 |
|  | *Ceratonia siliqua* | carob | Hellenistic-Roman | 1^st^ c. BCE | Zohary et al. 2012 |
|  | *Pinus pinea* | stone pine | Hellenistic-Roman | 1^st^ c. BCE | Kislev 1988 |
|  | *Prunus* subgen*. Cerasus/Prunus* | plum/cherry | Roman | 1^st^ c. CE | Tabak 2006 |
|  | *Pistacia vera* | pistachio | Roman | 2^nd^ c. CE | Hartman and Kislev 1998 |
|  | *Corylus sp.* | hazel | Roman | 2^nd^ c. CE | Kislev and Simchoni 2006; Langgut et al. 2021 |
|  | *Lupinus* *albus* | lupine | Early Islamic | 7^th^ c. CE | this paper |
|  | *Ziziphus jujuba/mauritiana* | jujube | Early Islamic | 7^th^ c. CE | this paper |
| IGR crop | *Solanum melongena* | aubergine | Early Islamic | 7^th^ c. CE | Amichay et al. 2019; this paper |

*Note: The earliest evidence for* Prunus *subgen.* Cerasus/Prunus *refers to plum (*Prunus *subgen.* Prunus*) only. Cherry (*Prunus *subgen.* Cerasus*) has yet to be identified in the southern Levantine archaeobotanical record.*

### References to Supplementary Table 7

Amichay, O., Ben-Ami, D., Tchekhanovets, Y., Shahack-Gross, R., Fuks, D. and Weiss, E. (2019) A bazaar assemblage: reconstructing consumption, production and trade from mineralised seeds in Abbasid Jerusalem. *Antiquity*, 93 (367): 199–217.

Caracuta, V., Vardi, J., Paz, Y. and Boaretto, E. (2017) Farming legumes in the pre-pottery Neolithic: New discoveries from the site of Ahihud (Israel). *PLOS ONE*, 12(5), e0177859.

Feldman, M. and Kislev, M.E. (2007) Domestication of emmer wheat and evolution of free-threshing tetraploid wheat. *Israel Journal of Plant Sciences*, 55(3-4), 207–221.

Hartman, A. and Kislev, M.E. (1998) Plant remains from the dwellers of the Ketef Yeriho caves at the end of the Bar-Kokhba revolt. In: Eshel, H., Amit, D. and Porat, R. (eds), Refuge caves of the Bar Kokhba revolt (pp. 153–168). Tel Aviv: Israel Exploration Society.

Kislev, M.E. (1988) *Pinus pinea* in agriculture, culture and cult. In: H. Küster, U. Körber-Gröhne, L. Baden-Württemberg (eds), *Der Prähistorische Mensch und seine Umwelt : Festschrift für Udelgard Körber-Grohne zum 65* Geburtstag (pp. 73–79). Stuttgart: Kommissionsverlag K. Theiss.

Kislev, M.E. (1990) Extinction of *Acacia nilotica* in Israel. In: Bottema, S., Entjes-Nieborg, G. and van Zeist, W. (eds), *Man’s role in the shaping of the Eastern Mediterranean landscape: Proceedings of the symposium on the impact of ancient Man on the landscape of the E Med Region & the Near East, Groningen, March 1989* (pp. 307–318). Groningen: CRC Press.

Kislev, M.E. and Simchoni, O. (2006) Botanical evidence for the arrival of refugees from Judea to refuge cave in Nahal Arugot in the fall of 135 CE. *Judea & Samaria Research Studies*, 15: 141–150. (Hebrew).

Kislev, M.E. and Simchoni, O. (2009). The secret of the good life at Orhan Mor (Moyat Awad) – a transit station along the Incense Route. *Judea & Samaria Research Studies* 12: 165–176. (Hebrew).

Kislev, M.E., Artzy, M. and Marcus, E. (1993) Import of an Aegean food plant to the middle bronze IIA coastal site in Israel. *Levant* 25(1): 145–154.

Langgut, D. (2015) Prestigious fruit trees in ancient Israel: first palynological evidence for growing *Juglans regia* and *Citrus medica*. *Israel Journal of Plant Sciences* *62*(1-2): 98–110.

Langgut, D., Cheddadi, R., Carrión, J.S., Cavanagh, M., Colombaroli, D., Eastwood, W. J., Greenberg R., Litt, T., Mercuri A.M., Miebach, A., Roberts, N., Woldring, H. and Woodbridge, J. (2019) The origin and spread of olive cultivation in the Mediterranean Basin: The fossil pollen evidence. *The Holocene*, *29*(5), 902–922.

Langgut, D., Tepper, Y., Benzaquen, M., Erickson-Gini, T. and Bar-Oz, G. (2021) Environment and horticulture in the Byzantine Negev Desert, Israel: Sustainability, prosperity and enigmatic decline. *Quaternary International*, 593–4: 160–177.

Melamed, Y. (2002) Chalcolithic and Hellenistic plant remains from Cave V/49 (Northern Judean Desert). ‘*Atiqot*, 41(2): 101–115.

Tabak, Y. (2006) *Agricultural prosperity in Roman Israel confirmed by Masada archeobotanic finds*. Unpublished PhD dissertation. Ramat-Gan: Bar-Ilan University.

Weiss, E. (2015) ‘Beginnings of fruit growing in the Old World’—two generations later. *Israel Journal of Plant Sciences*, 62(1–2): 75–85.

Weiss, E., Mahler-Slasky, Y., Melamed, Y., Lederman, Z., Bunimovitz, S., Bubel, S., and Manor, D. (2019). Foreign Food Plants as Prestigious Gifts: The Archaeobotany of the Amarna Age Palace at Tel Beth-Shemesh, Israel. *Bulletin of the American Schools of Oriental Research*, *381*(1): 83–105.

Zohary, D., Hopf, M. and Weiss, E. (2012) *Domestication of plants in the Old World*, 4th edition. Oxford: Oxford University Press.
