# Supplementary material for: Unprecedented yet gradual nature of first millennium CE intercontinental crop plant dispersal revealed in ancient Negev desert refuse
Source: eLife. 2023 Nov 27;12:e85118. doi: 10.7554/eLife.85118 (PMC10846859; doi:10.7554/eLife.85118)
Supplement: Supplementary file 8. [file elife-85118-supp8.docx]

Supplementary Table 8. Some *Acacia* spp. seed measurements from the Israel National Collection of Plant Seeds and Fruits

| **Species** | **Population** | **seed #** | **seed face (A/B)** | **seed length (mm)** | **seed width (mm)** | **areole length (mm)** | **areole width (mm)** | **(seed width-areole width)/ seed width** | **(seed length-areole length)/ seed length** | **max. areole width** |
| --- | --- | --- | --- | --- | --- | --- | --- | --- | --- | --- |
| *A. nilotica* | Elusa A, archaeological | 1 | A | 7.5 | 6.0 | 6.0 | 4.2 | 0.30 | 0.20 | a |
| *A. nilotica* | Elusa A, archaeological | 1 | B | 7.5 | 6.0 | 6.1 | 4.1 | 0.32 | 0.19 | a |
| *A. nilotica* | Elusa B, archaeological | 2 | A | 5.7 | 4.7 | 5.3 | 3.6 | 0.23 | 0.07 | a |
| *A. nilotica* | Luxor 1981 | 3 | A | 10.0 | 7.6 | 9.0 | 6.0 | 0.21 | 0.10 | a |
| *A. nilotica* | Luxor 1981 | 3 | B | 10.1 | 7.7 | 8.9 | 5.6 | 0.27 | 0.12 | a |
| *A. nilotica* | Luxor 1981 | 4 | A | 10.5 | 7.7 | 8.9 | 6.0 | 0.22 | 0.15 | a |
| *A. nilotica* | Luxor 1981 | 4 | B | 10.5 | 7.7 | 8.8 | 6.0 | 0.22 | 0.16 | a |
| *A. nilotica* | Luxor 1981 | 5 | A | 10.9 | 7.3 | 9.5 | 5.7 | 0.22 | 0.13 | a |
| *A. nilotica* | Luxor 1981 | 5 | B | 10.6 | 7.0 | 9.5 | 5.0 | 0.29 | 0.10 | a |
| *A. nilotica* | Luxor 1981 | 6 | A | 7.0 | 6.5 | 6.2 | 4.5 | 0.31 | 0.11 | a |
| *A. nilotica* | Luxor 1981 | 6 | B | 7.0 | 6.4 | 6.0 | 4.5 | 0.30 | 0.14 | a |
| *A. pachyceras* | Wadi Ram 26.2.95 | 7 | A | 9.2 | 6.9 | 6.2 | 3.2 | 0.54 | 0.33 | c |
| *A. pachyceras* | Wadi Ram 26.2.95 | 7 | B | 9.1 | 6.7 | 6.6 | 3.2 | 0.52 | 0.27 | c |
| *A. pachyceras* | Wadi Ram 26.2.95 | 8 | A | 10.5 | 8.0 | 7.4 | 4.2 | 0.48 | 0.30 | a |
| *A. pachyceras* | Wadi Ram 26.2.95 | 8 | B | 10.5 | 8.0 | 7.8 | 3.9 | 0.51 | 0.26 | a |
| *A. pachyceras* | Wadi Ram 26.2.95 | 9 | A | 10.6 | 6.5 | 7.9 | 3.5 | 0.46 | 0.25 | b |
| *A. pachyceras* | Wadi Ram 26.2.95 | 9 | B | 10.4 | 6.4 | 7.8 | 3.6 | 0.44 | 0.25 | b |
| *A. pachyceras* | Nahal Hayyun 15.3.71 | 10 | A | 8.1 | 5.7 | 5.0 | 2.7 | 0.53 | 0.38 | a |
| *A. pachyceras* | Nahal Hayyun 15.3.72 | 10 | B | 8.0 | 5.7 | 5.1 | 2.5 | 0.56 | 0.36 | a |
| *A. pachyceras* | Nahal Hayyun 15.3.73 | 11 | A | 8.5 | 5.8 | 6.4 | 3.3 | 0.43 | 0.25 | b |
| *A. pachyceras* | Nahal Hayyun 15.3.74 | 11 | B | 8.5 | 5.8 | 6.3 | 3.2 | 0.45 | 0.26 | b |
| *A. pachyceras* | Nahal Hayyun 15.3.75 | 12 | A | 7.7 | 6.2 | 6.0 | 3.6 | 0.42 | 0.22 | b |
| *A. pachyceras* | Nahal Hayyun 15.3.76 | 12 | B | 7.5 | 6.2 | 6.0 | 3.6 | 0.42 | 0.20 | b |
| *A. raddiana* | Moje Awad | 13 | A | 7.9 | 5.8 | 5.4 | 3.5 | 0.40 | 0.32 | e |
| *A. raddiana* | Moje Awad | 13 | B | 7.9 | 5.7 | 5.2 | 3.5 | 0.39 | 0.34 | e |
| *A. raddiana* | Moje Awad | 14 | A | 9.7 | 6.5 | 7.0 | 3.8 | 0.42 | 0.28 | d |
| *A. raddiana* | Moje Awad | 14 | B | 9.6 | 6.5 | 7.0 | 4.0 | 0.38 | 0.27 | c |
| *A. raddiana* | Moje Awad | 15 | A | 8.1 | 5.9 | 5.5 | 3.8 | 0.36 | 0.32 | c |
| *A. raddiana* | Moje Awad | 15 | B | 8.0 | 6.0 | 5.7 | 3.5 | 0.42 | 0.29 | c |
| *A. raddiana* | Ein Gedi 19.5.1917 | 16 | A | 8.0 | 5.5 | 5.9 | 3.5 | 0.36 | 0.26 | c |
| *A. raddiana* | Ein Gedi 19.5.1917 | 16 | B | 8.0 | 5.5 | 5.6 | 3.4 | 0.38 | 0.30 | c |
| *A. raddiana* | Ein Gedi 19.5.1917 | 17 | A | 8.0 | 5.3 | 5.9 | 3.5 | 0.34 | 0.26 | c |
| *A. raddiana* | Ein Gedi 19.5.1917 | 17 | B | 8.1 | 5.4 | 5.5 | 3.4 | 0.37 | 0.32 | c |
| *A. raddiana* | Ein Gedi 19.5.1917 | 18 | A | 8.0 | 5.4 | 5.9 | 3.5 | 0.35 | 0.26 | c |
| *A. raddiana* | Ein Gedi 19.5.1917 | 18 | B | 8.0 | 5.4 | 5.8 | 3.4 | 0.37 | 0.28 | c |

### Table uses Acacia as used in the reference accessions; for synonyms see text above. Max. areole width is based on distance from hilum: a) upper third (from hilum); b) upper third-midway; c) midway; d) midway-lower third; e) lower third
