# Supplementary material for: Unprecedented yet gradual nature of first millennium CE intercontinental crop plant dispersal revealed in ancient Negev desert refuse
Source: eLife. 2023 Nov 27;12:e85118. doi: 10.7554/eLife.85118 (PMC10846859; doi:10.7554/eLife.85118)
Supplement: Supplementary file 9. [file elife-85118-supp9.docx]

Supplementary Table 9. Select *L. clymenum* seed measurements from Tel Nami

| Seed | Length (mm) | Breadth (mm) | Thickness (mm) | Length/Breadth | Length/Thickness |
| --- | --- | --- | --- | --- | --- |
| 1 | 4.3 | 2.3 | 3.6 | 1.87 | 1.19 |
| 2 | 4.6 | 2.4 | 3.9 | 1.92 | 1.18 |
| 3 | 4.2 | 2.2 | 3.05 | 1.91 | 1.38 |
| 4 | 3.6 | 2.75 | 2.9 | 1.31 | 1.24 |
| 5 | 3.3 | 2.5 | 3.5 | 1.32 | 0.94 |
| mean | 4.00 | 2.43 | 3.39 | 1.66 | 1.19 |
| s.d. | 0.48 | 0.19 | 0.37 | 0.29 | 0.14 |
